# Chlorophyll treatment combined with photostimulation increases glycolysis and decreases oxidative stress in the liver of type 1 diabetic rats

**DOI:** 10.1590/1414-431X20198389

**Published:** 2019-12-20

**Authors:** A.L.M. Wunderlich, S.C.S.F. Azevedo, L.A. Yamada, C. Bataglini, C. Previate, K.S.S. Campanholi, P.C.S. Pereira, W. Caetano, V. Kaplum, C.V. Nakamura, A.B.S. Nakanishi, J.F. Comar, M.M.D. Pedrosa, V.A.F. Godoi

**Affiliations:** 1 Programa de Pós-Graduação em Ciências Fisiológicas, Universidade Estadual de Maringá, Maringá, PR, Brasil; 2 Programa de Pós-Graduação em Ciências Biológicas, Universidade Estadual de Maringá, Maringá, PR, Brasil; 3 Programa de Pós-Graduação em Química, Universidade Estadual de Maringá, Maringá, PR, Brasil; 4 Departamento de Química, Universidade Estadual de Maringá, Maringá, PR, Brasil; 5 Programa de Pós-Graduação em Ciências Farmacêuticas, Universidade Estadual de Maringá, Maringá, PR, Brasil; 6 Departamento de Ciências Básicas da Saúde, Universidade Estadual de Maringá, Maringá, PR, Brasil; 7 Departamento de Bioquímica, Universidade Estadual de Maringá, Maringá, PR, Brasil; 8 Departamento de Ciências Fisiológicas, Universidade Estadual de Maringá, Maringá, PR, Brasil

**Keywords:** Photopigment, Diabetes, Metabolism, Free Radicals, Phototherapy

## Abstract

Photodynamic therapy (PDT) promotes cell death, and it has been successfully employed as a treatment resource for neuropathic complications of diabetes mellitus (T1DM) and hepatocellular carcinoma. The liver is the major organ involved in the regulation of energy homeostasis, and in pathological conditions such as T1DM, changes in liver metabolic pathways result in hyperglycemia, which is associated with multiple organic dysfunctions. In this context, it has been suggested that chlorophyll-*a* and its derivatives have anti-diabetic actions, such as reducing hyperglycemia, hyperinsulinemia, and hypertriglyceridemia, but these effects have not yet been proven. Thus, the biological action of PDT with chlorophyll-*a* on hepatic parameters related to energy metabolism and oxidative stress in T1DM Wistar rats was investigated. Evaluation of the acute effects of this pigment was performed by incubation of isolated hepatocytes with chlorophyll-*a* and the chronic effects were evaluated by oral treatment with chlorophyll-based extract, with post-analysis of the intact liver by *in situ* perfusion. In both experimental protocols, chlorophyll-*a* decreased hepatic glucose release and glycogenolysis rate and stimulated the glycolytic pathway in DM/PDT. In addition, there was a reduction in hepatic oxidative stress, noticeable by decreased lipoperoxidation, reactive oxygen species, and carbonylated proteins in livers of chlorophyll-treated T1DM rats. These are indicators of the potential capacity of chlorophyll-*a* in improving the status of the diabetic liver.

## Introduction

Diabetes mellitus is a serious chronic metabolic disorder that affects more than 415 million people worldwide, being one of the leading causes of death in Brazil in 2015 (1). In type 1 diabetes mellitus (T1DM), hyperglycemia – the most prominent feature of this pathology – is a result of both the impaired glucose uptake by insulin-dependent tissues and the hyperactive production of glucose by the liver, due to the lack of insulin release by pancreatic beta cells. This impaired glucose homeostasis is associated with multiple organic dysfunctions and metabolic abnormalities in the liver pathways of blood glucose regulation, such as reduction of the enzymatic activity of the glycolytic and glycogen-synthesizing pathways and increase of the gluconeogenic enzymes (2).

In addition to indispensable insulin therapy, there are several non-medication interventions that can be used as adjuvants in the treatment of T1DM (3), but they do not hinder the progression of chronic complications and, therefore, the search for new therapeutic strategies is necessary. Among different interventions, photodynamic therapy (PDT) has been prominent in the treatment of neoplastic diseases (4–6) and neuropathic complications of T1DM (7). This therapy consists of topical or systemic administration of a photosensitizing agent (PS) and its accumulation in the diseased tissue followed by local irradiation with light at a wavelength within the PS absorption spectrum (8). With photoexcitation, the PS can react with oxygen and generate singlet oxygen (^1^O_2_) and reactive oxygen species (ROS) (9), provoking cytotoxicity and photodamage, that lead affected cells to death (6).

Due to economic and environmental considerations, PSs obtained from abundant raw materials attract greater interest than those prepared by complex chemical procedures (10). Among raw materials, chlorophyll (Chl) is the most abundant plant photopigment in nature, with chlorophyll-*a* (Chl*-a*) corresponding to approximately 75% of the green pigments found in plants (11). Structurally, Chl*-a* is a fully unsaturated asymmetric macrocyclic molecule with a hydrophobic character (11) that gives it low solubilization in hydrophilic solutions. Therefore, its incorporation into micellar copolymers, such as P123, which has been proven to be biocompatible, is necessary for *in vivo* and *in vitro* analyses, since they guarantee the monomerization of the hydrophobic PS and the maintenance of its photophysical properties, which are indispensable for PDT (12).

Chl*-a* metabolites are retinoic X receptor (RXR) agonists (13). Research has shown that these agonists are capable of decreasing hyperglycemia, hyperinsulinemia, and hypertriglyceridemia in type 2 diabetic mice (T2DM) (14). Therefore, it has been suggested that Chl*-a* metabolites could also exert such anti-diabetic actions (15). However, the physiological potential of this pigment and its role in the prevention of chronic complications of diabetes have been neglected (16).

Evidence of the accumulation of Chl*-a* and its metabolites in organs such as intestine and liver (17,18) suggests that these organs might be affected by these compounds. Considering that liver functioning is compromised in the absence of endogenous insulin in T1DM, that hyperglycemia and oxidative stress are relevant in this disease, and that Chl-*a* may have beneficial effects, the hypothesis formulated was that Chl-*a* and/or its metabolites could have important anti-diabetic effects on this model. Thus, the purpose of this study was to evaluate the biological action of PDT, applied to Chl*-a*, on the hepatic parameters related to energy metabolism and oxidative stress in T1DM rats.

## Material and Methods

### Chlorophyll-based extract and chlorophyll-*a* incorporated into P123 micellar nanostructured system

**Figure 1:**
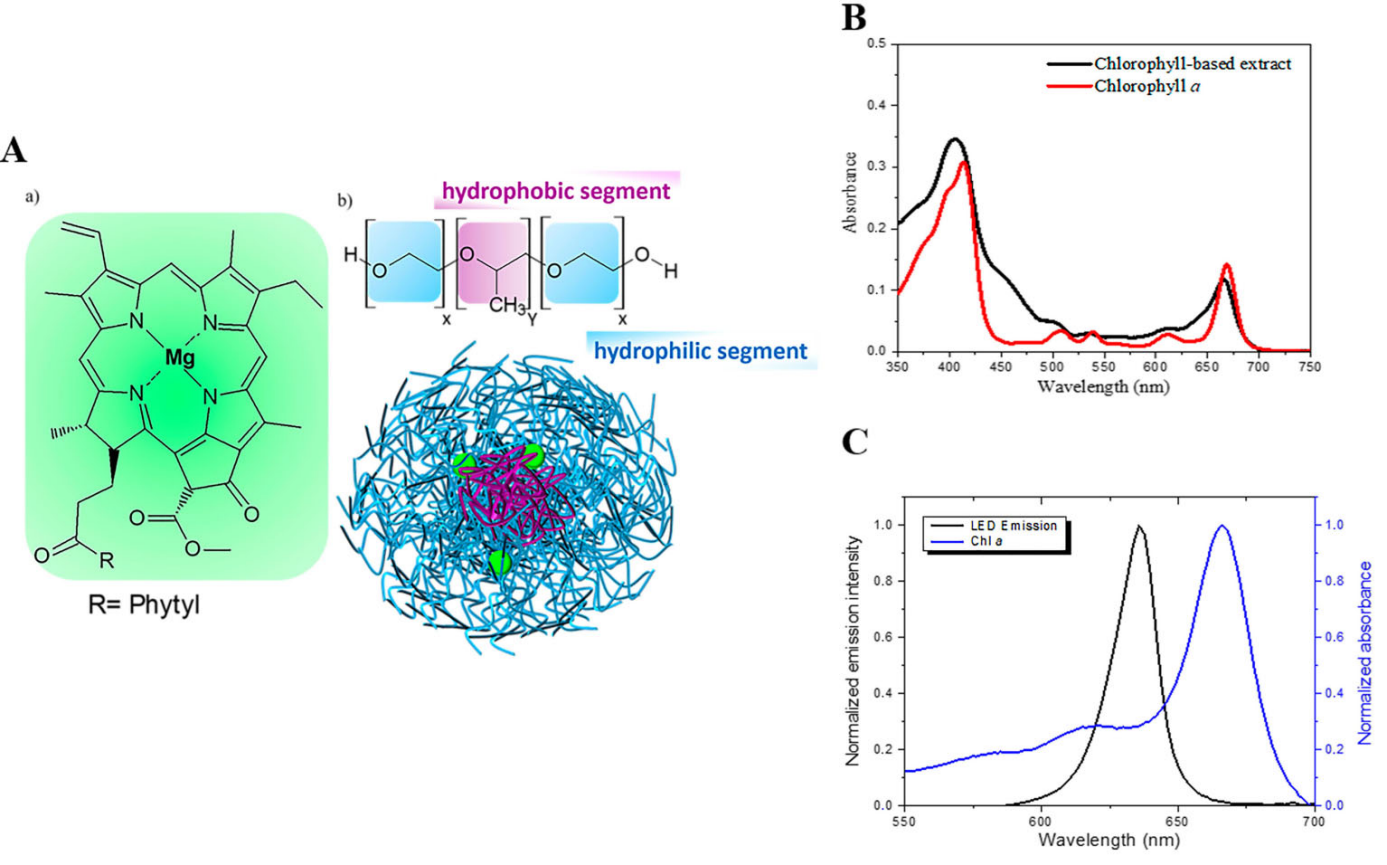
Schematic representation of the encapsulation of chlorophyll-*a* (Chl*-a*) into a P123 micelle and its absorption spectra. **A**, Chemical structure of (**a**) Chl*-a* in inclusion to (**b**) P123 triblock copolymer (x=20 and y=70). **B**, UV-Vis absorption spectra for Chl*-a* (1.25 mg/L) and chlorophyll-based extract (22 mg/L) incorporated into the 2% (m/v) P123 micellar system. **C**, Normalized absorption spectra UV-Vis (Chl*-a* in P123) superimposed to the red light-emission diode (LED).

The chlorophyll-based extract (CBE) and purified chlorophyll-*a* were obtained through the methodology proposed by Campanholi et al. (19). The concentration of CBE (22 mg/L) and Chl*-a* (1.25 mg) in the micellar copolymer P123 (2% m/v) were determined by the solid dispersion method (20), which consists in co-solubilizing the drug and P123 in ethanol, followed by evaporation of the solvent and formation of a thin film. This solid matrix was maintained under vacuum for 12 h and then hydrated with Krebs/Henseleit-bicarbonate (KH) buffer solution, pH 7.6, at 60°C, under vigorous stirring. The same procedure was performed without the addition of the active principle (CBE or Chl*-a*) in order to evaluate the individual effect of the P123 copolymer. The doses of Chl*-a* and CBE administered in the experimental protocols were determined by the Research Group in Photodynamic Systems (NUFESP) of the Department of Chemistry (State University of Maringá, Brazil), responsible for the development and production of the compounds (21). The lighting system used in the studies was composed of light-emitting surface-mounted diodes that emitted red light with a maximum wavelength of 636 nm. The photonic standardization of the light source was made in an Ocean Optics USSB 2000+ photo-radiometer (USA) and the measured irradiance was of 2.57 10^3^ µWatts/cm^2^. Its emission spectrum ensured overlap with the absorption spectrum of the photosensitizers (Figure 1). The lighting system was maintained at a fixed distance of 10 cm in all tests.

### Animals and induction of type 1 diabetes mellitus

Adult male Wistar rats (40 animals, 90 days old, 200 g body weight) were kept at the animal house under controlled temperature (23–25°C), photoperiod (12 h light/12 h dark), and they were given standard rodent chow (Nuvital^®^, Nuvilab, Brazil) and water *ad libitum*. All the procedures were approved by the Ethics Commission on Animal Use of the State University of Maringá (CEUA 2019130116).

Diabetes induction consisted of intravenous injection of streptozotocin (60 mg/kg body weight; Sigma, USA) dissolved in citrate buffer (10 mM, pH 4.5, final volume 0.1 mL/100 g bw) after overnight fasting (control animals received citrate buffer, 10 mM, pH 4.5, final volume 0.1 mL/100 g bw). The confirmation of the diabetic state was made one week later by checking the fasting glycemia. Animals with glycemia ≥300 mg/dL were considered diabetic.

### Experimental design

Control and diabetic animals were randomly allocated to one of the following groups: CG, control rats without light irradiation; CG/PDT, control rats intended for treatment with light irradiation; DM, type 1 diabetic rats without light irradiation; and DM/PDT, diabetic rats with light irradiation. The following procedures were carried out (5 animals per group per procedure): hepatocyte incubation, *in vivo* biodistribution, and *in situ* liver perfusion. For all protocols, the animals were in a fed state, to allow the evaluation of glycogenolytic and glycolytic pathways.

### Incubation of hepatocytes

To evaluate the acute effect of PDT with Chl*-a*, hepatocytes were isolated through the previously described 3% collagenase perfusion technique (22). Hepatocytes with viability above 75% (10^6^ cells/mL) were incubated in oxygenated (O_2_/CO_2_ 95/5%) KH buffer pH 7.4, 37°C, under constant stirring for 1 h in the absence (pure KH or empty P123 micellar copolymer) or presence of Chl*-a* in the P123 micellar copolymer at the concentration of 1.25 mg/L. The hepatocytes of CG and DM groups were incubated in the dark, while those from CG/PDT and DM/PDT groups were incubated with red light irradiation. After this period, the samples were centrifuged three times at 426 *g* for 4 min at 4°C, and the soluble fraction collected for determination of glucose, L-lactate, and pyruvate. The results are reported as μmol·10^6^ cells^-1^·h^-1^.

### *In vivo* biodistribution

To evaluate the biodistribution of Chl*-a*, five animals from CG and DM groups were treated orally, by gavage, with CBE for 14 consecutive days at a concentration of 22 mg/L (corresponding to the profile and intensity of electronic absorption of Chl*-a* used in the incubation medium of the isolated hepatocytes; Figure 1) in a volume of 0.4 mL per animal. During the treatment period, food and water intake, fasting glycemia, and body weight were recorded.

Just after the CBE treatment, animals were evaluated for the *in vivo* biodistribution of the pigments contained in the CBE. They were anesthetized (thiopental 40 mg/kg bw and lidocaine 5 mg/kg bw, *ip*, 0.1 mL/100 g bw), had their abdominal fur removed, and then evaluated using MS FX PRO (Carestream Molecular Imaging, Carestream Health, USA). Fluorescence images (excitation=650 nm; emission=700 nm) were obtained with a CCD camera (Kodak Image Station, Canada) 1, 2, and 24 h after gavage with CBE. The images were acquired by Carestream Molecular Imaging 5.0.

### *In situ* liver perfusion

Based on the results of the hepatocyte incubation protocol, five rats from the CG, DM, and DM/PDT groups were treated orally by gavage with CBE 22 mg/L, 0.4 mL per animal for 14 consecutive days and then submitted to *in situ* liver perfusion in order to evaluate the chronic effect of Chl*-a* PDT.

After 14 days, the fed animals were anesthetized (thiopental 40 mg and lidocaine 5 mg/kg bw, *ip*, 0.1 mL/100 g bw) and had the liver perfused with KH buffer, oxygenated by carbogenic mixture (O_2_/CO_2_ 95/5%), pH 7.4, 37°C, at a flow rate calculated from the body weight and adjusted to values that allowed adequate oxygenation (4 mL·min^-1^·g liver^-1^). The organ was completely exsanguinated and euthanasia occurred by hypovolemic shock.

The KH entered the liver through the portal vein and exited through the cava vein in an open and non-recirculating perfusion system. Perfused samples were collected every 5 min for 1 h for biochemical determinations. The first 15 min were considered baseline perfusion and the next 45 min stimulated perfusion, during which the liver was kept in the dark (CG and DM) or irradiated with red light (DM/PDT). Thus, all groups had the initial 15 min to release glycogen stores without any stimulation.

### Blood and biochemical parameters

The fluid collected during the perfusion was used to evaluate the concentration of glucose, L-lactate, and pyruvate. The data are reported as area under the curve (AUC, μmol/g liver). In addition, the hepatic uptake of the CBE was evaluated by spectrophotometric measurements of electronic absorption and fluorescence emission for each time point of the perfusion.

Blood samples were collected by cardiac puncture immediately after the anesthesia of the animals of the liver perfusion. Hepatic injury was determined by the plasma concentration of alanine amino transferase (ALT) and aspartate amino transferase (AST). The kits for biochemical analysis were purchased from Gold Analisa^®^ Diagnóstica Ltda. (Brazil).

### Preparation of liver homogenate and analytical procedures

At the end of the perfusion, still in the metabolic steady state, the liver was removed, rapidly clamped, and stored in liquid nitrogen. Later, a sample of the organ was homogenized in van Potter Elvejem homogenizer (Sigma Chemical Co., USA) with 10 volumes of 0.1 M potassium phosphate buffer (pH 7.4). An aliquot of this homogenate was used to determine the following parameters: levels of carbonylated proteins, lipoperoxides, and content of reduced and oxidized glutathione. The remaining homogenate was centrifuged at 20,000 *g* for 20 min at 4°C and the supernatant used to determine the activity of the antioxidant enzymes catalase and superoxide dismutase, as well as the content of ROS. Protein content of total homogenate and supernatant were determined as previously described (23).

### Carbonylated proteins

The levels of carbonyl groups in proteins were determined by spectrophotometry with 2,4-dinitrophenylhydrazine (DNPH) (ε_370_=22·10^3^ M^-1^·cm^-1^) (24) and values are reported as nmol/mg protein.

### Lipoperoxides

The levels of lipid peroxidation were evaluated by thiobarbituric acid reactive substances (TBARS) (25). The amount of TBARS was calculated from the standard curve prepared with 1,1′,3,3′-tetraethoxypropane and the values are reported as nmol/mg protein.

### Reduced (GSH) and oxidized glutathione (GSSG)

The contents of GSH and GSSG were determined by spectrofluorometry (excitation at 350 nm and emission at 420 nm) by the o-phthalaldehyde (OPT) assay (26). Fluorescence was estimated as GSH. For the GSSG assay, the sample was pre-incubated with 10 mM n-ethylmaleimide (NEM) and then with a solution containing 1M NaOH and OPT. The results were calculated using a standard curve prepared with GSH or GSSG and values are reported as nmol/mg protein.

### Reactive oxygen species

ROS levels were quantified by spectrofluorometry via 2,7-dichlorofluorescein diacetate (DCFH-DA) as described previously (27). This method quantifies the conversion of DCFH-DA to the oxidized and fluorescent molecule 2,7-dichlorofluorescein (DCF) in the presence of esterases and ROS. The results are reported as nmol/mg protein using a standard curve prepared with DCF.

### Activity of antioxidant enzymes: catalase and superoxide dismutase (SOD)

Catalase activity was estimated by measuring changes in absorbance at 240 nm using H_2_O_2_ as the substrate (28), which are reported as μmol·min^-1^·mg protein^-1^. The activity of SOD was estimated by its ability to inhibit the auto-oxidation of pyrogallol in alkaline medium, which was determined by spectrophotometry at 420 nm (29). A SOD unit is considered the amount of enzyme that is capable of causing 50% inhibition, and the results are reported as U/mg protein.

### Statistical analysis

Data were submitted to Kolmogorov-Smirnov test to verify normality. One-way analysis of variance (ANOVA) with Tukey *post hoc* test was used to compare the groups, and the level of significance was pre-fixed at 95% (P<0.05), using Prism version 5.0 (GraphPad, USA). The results are reported as means±SD.

## Results

### Isolated hepatocytes

**Figure 2:**
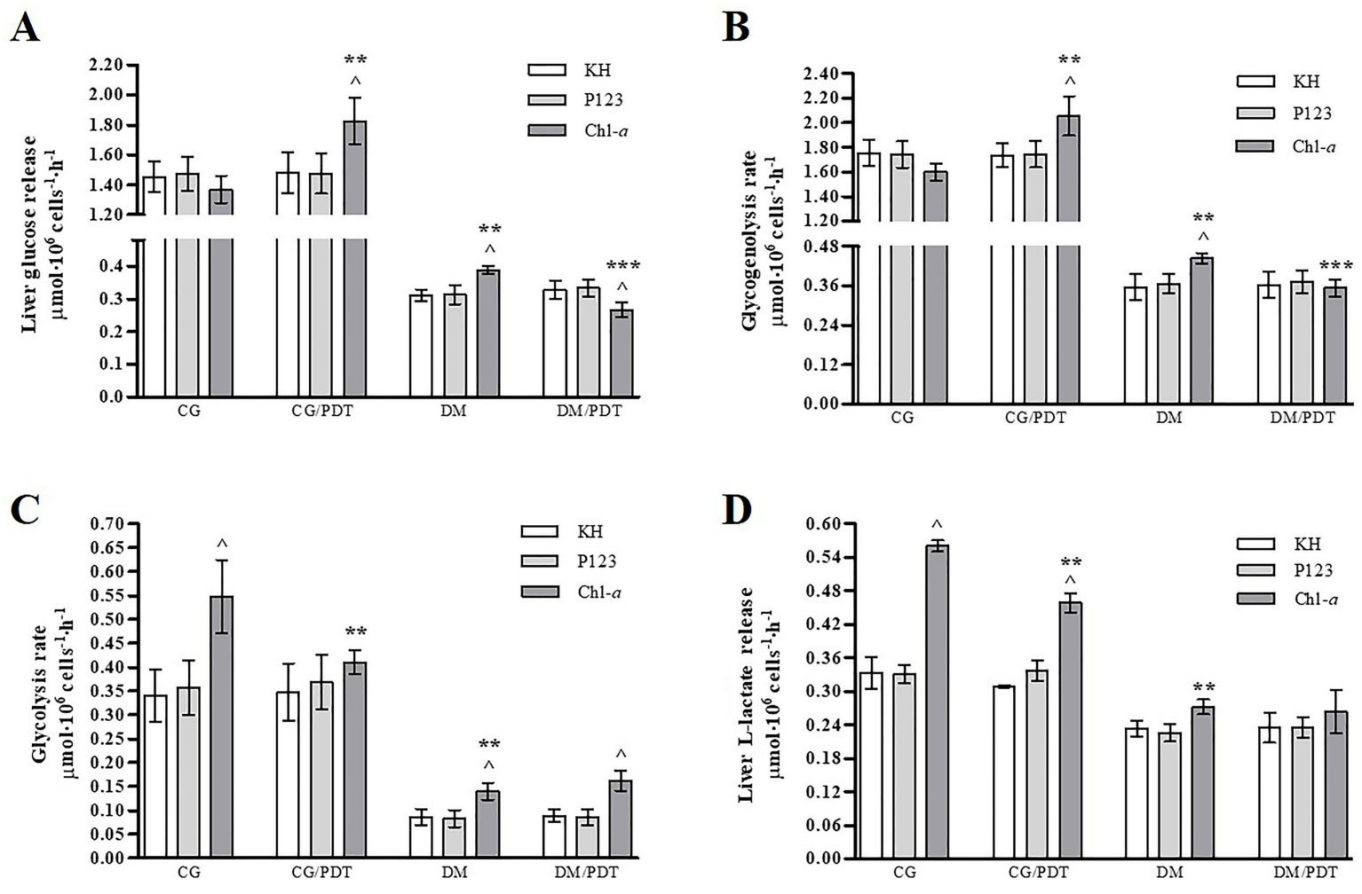
Glucose release (**A**), glycogenolysis (**B**), glycolysis rates (**C**), and L-lactate release (**D**) from isolated hepatocytes of Wistar rats. KH: hepatocytes incubated with Krebs/Henseleit-bicarbonate buffer; P123: hepatocytes incubated with empty micellar copolymers; Chl*-a*; hepatocytes incubated with chlorophyll-*a* (1.25 mg/L). CG: control animals without light irradiation; CG/PDT: control animals with light irradiation; DM: type 1 diabetic animals without light irradiation; DM/PDT: diabetic animals with light irradiation. Data reported as means±SD, n=5/group. âP<0.05 *vs* KH and P123 of the same group, **P<0.05 *vs* Chl*-a* CG, ***P<0.05 *vs* Chl*-a* DM (one-way ANOVA/Tukey).

Hepatocytes were incubated in the presence of Chl*-a* and controls were run in parallel, in which the hepatocytes were incubated only with KH buffer or with empty P123 micellar copolymers. Glucose and L-lactate release and rates of glycogenolysis and glycolysis of liver cells are presented in Figure 2.

The results showed the absence of biological response to the empty micellar copolymers, given the equivalence with the results obtained from the incubation of hepatocytes in the presence of KH buffer only. It was also observed that, in general, all metabolic parameters presented in Figure 2 were decreased in DM compared to CG.

In the dark, Chl*-a* increased the rate of glycolysis and L-lactate production from CG hepatocytes, without altering glucose release and the glycogenolysis rate. In DM, glucose release, glycogenolysis, and glycolysis were stimulated by Chl*-a*. In the PDT condition, Chl*-a* also increased glucose release and rates of glycogenolysis and glycolysis in CG/PDT (*vs* CG), whereas in DM/PDT hepatocytes there was stimulation of glycolysis only, accompanied by reduction of glucose release and glycogenolysis (*vs* DM). The concentration of pyruvate in the supernatant of the isolated hepatocytes was below the lower limits of detection of the method; therefore, the cytosolic NADH/NAD^+^ ratio could not be calculated.

### Parameters related to the treatment with chlorophyll-based extract

The goal of the second part of the experiments was to evaluate the effect of Chl*-a in natura* on plasmatic parameters, hepatic metabolism, and oxidative stress in the intact liver. For this, CBE was administered orally (gavage) for 14 days. During the treatment period, the classic symptoms of diabetes were observed in the DM groups, represented by reduced body mass and increased fasting glycemia compared to CG, both before (not shown) and after (Table 1) gavage with CBE. It was also observed, by comparing the groups before and after the gavage period, that these parameters were not affected by the treatment. There was also no change in water and food intake (data not shown) promoted by treatment – in addition to those caused by T1DM itself (polydipsia and polyphagia).

**Table 1:** Body weight, fasting glycemia, and plasma markers of hepatic injury of Wistar rats after 14 days of oral administration of chlorophyll-based extract (CBE; 22 mg/L) or saline.

	Saline		CBE	
	CG	DM	CG	DM
Body weight (g)	403.70±8.43	318.40±6.85^*^	442.20±5.46	317.80±12.65^*^
Fasting glycemia (mg/dL)	87.60±5.24	385.80±10.23^*^	91.33±1.86	473.70±8.56^*^
AST (U/L)	50.38±1.06	181.50±7.81^*^	38.90±0.93	189.00±16.90^*^
ALT (U/L)	22.43±1.76	198.5±8.51^*^	31.80±1.77	205.30±15.23^*^

Data reported as means±SD, n=5/group. CG: control animals; DM: type 1 diabetic animals; AST: aspartate amino transferase; ALT: alanine amino transferase. ^*^P<0.05 DM *vs* CG (one-way ANOVA/Tukey).

**Figure 3:**
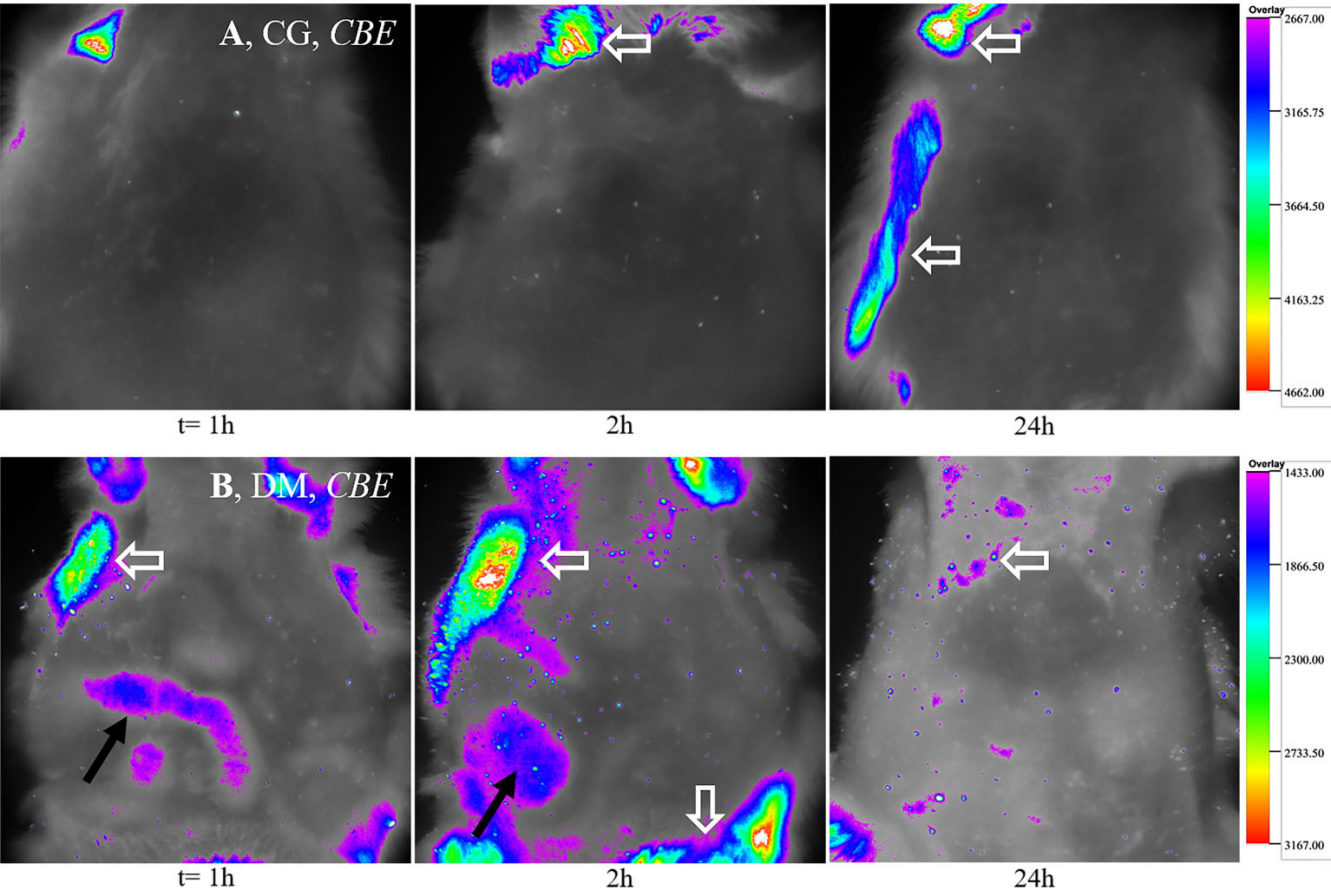
*In vivo* images of the abdominal region of Wistar rats from control animals (CG) (**A**) and type 1 diabetic animals (DM) (**B**) groups. CBE: chlorophyll-based extract. The intensity of the body fluorescence, presented as RLU/pixels per 30 s, was evaluated at different times (h) after gavage. The narrow black arrows signal the fluorescence emission of the compound present in the intestinal loop of the animals. The broad white arrows represent fluorescence artifacts produced by the hairs of animals, as well as other unlabeled fluorescent sites. n=5/group.

The biodistribution analysis of the photosensitive components present in the CBE, shown in Figure 3, revealed the absence of fluorescence emission – referring to CBE components – in any body region in non-diabetic animals after 14 days of gavage treatment. On the other hand, fluorescent emission in the intestinal loops of diabetic animals was observed 1 h after its administration by gavage. The fluorescence intensity in the intestine was reduced after 2 h and absent 24 h after administration of the CBE.

The evaluation of hepatic injury by the quantification of AST and ALT showed a significant increase in both diabetic groups (DM and DM/PDT) compared to non-diabetic animals. In order to investigate whether hepatic lesion was a result of disease or treatment with CBE, non-diabetic and diabetic animals were submitted to the same oral treatment protocol, but with saline administration. The results confirmed the increased AST and ALT in diabetic animals, regardless of the oral treatment (Table 1).

### *In situ* liver perfusion

**Figure 4:**
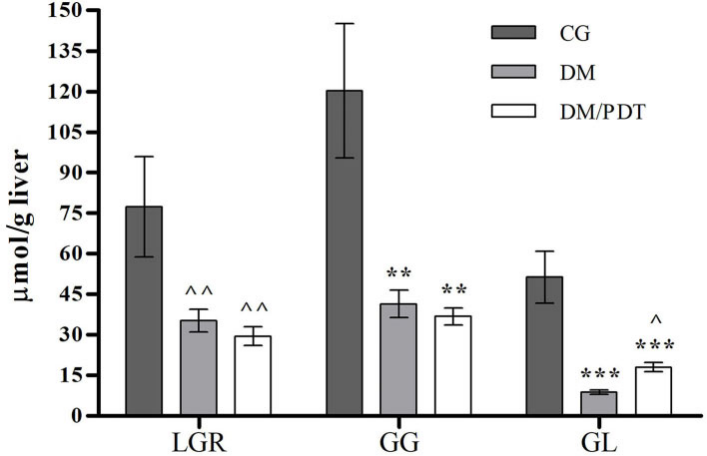
Liver glucose release (LGR), glycogenolysis (GG), and glycolysis (GL) rates during *in situ* perfusion of the liver of Wistar rats. CG: control animals without light irradiation; DM: type 1 diabetic animals without light irradiation; DM/PDT, diabetic animals with light irradiation. Data reported as means±SD, n=5/group. ââP<0.05 *vs* LGR of CG, **P<0.05 *vs* GG of CG, ***P<0.05 *vs* GL of CG, âP<0.05 *vs* GL of DM (one-way ANOVA/Tukey).

Figure 4 shows AUC values of liver glucose release and the hepatic rates of glycogenolysis and glycolysis. The results indicated reduction in all of these parameters in the diabetic groups (DM and DM/PDT) compared to CG, in the order of 50–60% for liver glucose release, 60–70% for glycogenolysis, and 60–80% for glycolysis. In addition, the reduction in liver glucose release and glycogenolysis rate did not differ between the diabetic groups. But with irradiation, DM/PDT had a higher glycolysis rate than DM.

Similar to previous results, production of L-lactate and pyruvate during liver perfusion (Table 2) was lower in both diabetic groups compared to CG. However, DM/PDT animals had higher L-lactate production accompanied by lower production of pyruvate than DM, which agreed with the glycolytic stimulation in this group. The cytoplasmic NADH/NAD^+^ ratio also differed between the diabetic groups, and DM/PDT had a higher ratio compared to the other groups.

**Table 2:** Production of L-lactate and pyruvate, and the cytosolic NADH/NAD^+^ ratio of the perfused liver of Wistar rats after 14 days of oral administration of chlorophyll-based extract (CBE; 22 mg/L).

	CG	DM	DM/PDT
L-Lactate (µmol/g liver)	30.82±2.39	7.70±0.43^*^	18.07±1.26^*^â
Pyruvate (µmol/g liver)	13.73±0.09	1.32±0.07^*^	0.39±0.16^*^â
NADH/NAD^+^ ratio	2.79±0.40	6.63±0.69	45.63±3.20^*^â

Data reported as means±SD, n=5/group. CG: control animals without light irradiation; DM: type 1 diabetic animals without light irradiation; DM/PDT: diabetic animals with light irradiation. ^*^P<0.05 *vs* CG, âP<0.05 *vs* DM (one-way ANOVA/Tukey).

The spectrophotometric measurements of electronic absorption and fluorescence emission showed the absence of extract in the effluent fluid collected during liver perfusion, suggesting that it was completely taken up by the organ.

### Hepatic oxidative stress

**Figure 5:**
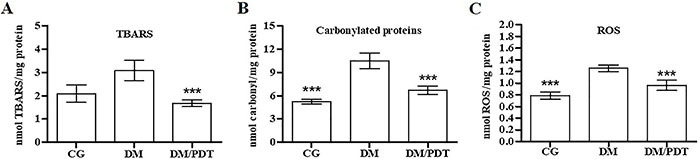
Hepatic oxidative stress of Wistar rats after 14 days of oral administration of chlorophyll-based extract (CBE; 22 mg/L). Levels of thiobarbituric acid reactive substances (TBARS) (**A**) and carbonylated proteins (**B**) in liver total homogenate, and reactive oxygen species (ROS) (**C**) in the supernatant of total liver homogenate after 1 h of infusion with KH buffer of fed Wistar rats. CG: control animals without light irradiation; DM: type 1 diabetic animals without light irradiation; DM/PDT: diabetic animals with light irradiation. Data reported as means±SD, n=5/group. ***P<0.05 *vs* DM (one-way ANOVA/Tukey).

The levels of TBARS, carbonylated proteins, and ROS in non-diabetic (CG) and diabetic (DM and DM/PDT) animals treated with CBE for 14 days and submitted to the infusion protocol with KH for 1 h are shown in Figure 5. These parameters of oxidative stress were elevated in DM compared to CG, suggesting that the oxidative characteristic of T1DM was not altered by oral treatment with CBE alone. On the other hand, PDT in the liver of diabetic animals (DM/PDT) reduced the hepatic TBARS levels by 46%, carbonylated proteins by 36%, and ROS by 25% compared to DM, statistically matching the values of DM/PDT to CG. These results suggested that PDT, during the *in situ* liver perfusion, was able to alter these parameters of hepatic oxidative stress of diabetic animals. Given this alteration, the antioxidant system was also evaluated in the perfused liver through determinations of GSH and GSSG levels and the activity of the catalase and SOD enzymes (Table 3).

**Table 3:** Activity of the antioxidant system on total liver homogenates of Wistar rats after 14 days of oral administration of chlorophyll-based extract (CBE; 22 mg/L).

	CG	DM	DM/PDT
GSH (nmol/mg protein)	8.644±0.630	13.332±0.953^*^	11.270±0.793
GSSG (nmol/mg protein)	0.856±0.062	0.822±0.023	1.387±0.096^*^â
GSH/GSSG	10.133±0.560	13.179±0.900^*^	8.291±0.952â
GSH + 2x GSSG (nmol GSH/mg protein)	10.357±0.725	14.978±0.984^*^	14.045±0.741^*^
Catalase (µmol·min^−1^·mg protein^−1^)	981.70±12.00	495.39±35.60^*^	773.26±101.38â
SOD (U/mg protein)	2.56±0.17	1.54±0.08^*^	1.82±0.14^*^

Data reported as means±SD, n=5/group. CG: control animals without light irradiation; DM: type 1 diabetic animals without light irradiation; DM/PDT: diabetic animals with light irradiation. GSH: reduced glutathione; GSSG: oxidized glutathione; SOD: superoxide dismutase. ^*^P<0.05 *vs* CG, âP<0.05 *vs* DM (one-way ANOVA/Tukey).

In the dark, livers of diabetic animals had high levels of GSH compared to CG, while PDT raised GSSG levels by approximately 70% compared to other groups. High GSH levels in DM and GSSG in DM/PDT caused approximately 40% increase in total glutathione levels in both groups relative to CG. The GSH/GSSG ratio was 30% higher in DM (relative to CG) and 38% lower in DM/PDT compared to DM.

Catalase activity in DM was only half that of CG and increased by 56% in the presence of the light stimulus. In both diabetic groups (DM and DM/PDT), SOD enzyme activity was lower compared to CG.

## Discussion

Despite many existing studies in the field of PDT (9), its applicability and/or effect on internal organs in *in vivo* models remain undetermined and, therefore, no important descriptive patterns were found in the literature to compare the findings of this study.

This research tested the hepatic effects of PDT with the PS Chl*-a* in untreated T1DM rats. The results showed that T1DM affected both the cellular metabolism and the response of hepatocytes to PS. The results suggested that in CG, Chl*-a* increased the intracellular consumption of glucose by stimulating glycolysis, which could then have been converted to L-lactate, whose production was high. In CG/PDT, the action of Chl*-a* stimulated the release of glucose, glycogenolysis, and L-lactate production, but did not affect glycolysis. In the diabetic condition, in the dark (DM), unlike what was observed in hepatocytes of healthy animals, Chl*-a* altered the metabolic response of liver cells, with glucose release, glycogenolysis, and glycolysis being stimulated. With photoexcitation (DM/PDT), Chl*-a* decreased the release of glucose and glycogenolysis and maintained the stimulation of the glycolytic pathway. Thus, in the model of interest (DM/PDT), PDT with Chl*-a* induced an adequate response to the pathological picture.

Metabolic results obtained from *in situ* liver perfusion showed that liver glucose release, glycogenolysis, and glycolysis were decreased in both diabetic groups (illuminated or not) relative to CG. This is because in T1DM the absence of insulin decreases the activity of the glycolytic and glycogen-synthesizing enzymes (2) and, therefore, glucose utilization and metabolism become reduced (30). However, there was no difference between the results obtained through *in situ* liver perfusion between the non-illuminated (DM) and the illuminated (DM/PDT) diabetic groups, except for the glycolysis rate, showing that they appear to be independent of the photo-excitatory condition.

Comparison of the results obtained with isolated hepatocytes (acute Chl*-a*) and with the intact organ (Chl*-a* chronic effect) of CG animals revealed that in isolated cells there was stimulation only of the glycolytic pathway, while in the intact liver glucose release, glycogenolysis and glycolysis rates were elevated. It is possible that the wider effect of the PS in the experiment with the intact organ is due to the action of Chl*-a* and its metabolites produced after gastric digestion, once the degradation of Chl*-a* by gastric acid and the absorption of its metabolites as micelles by the enterocytes were already demonstrated (11,16). However, considering the absence of fluorescence (Figure 3) after 14 days of treatment with CBE, it is likely that the pigments were not incorporated by the intestine of the healthy organism and thus the liver of the non-diabetic animal remained at the expected physiological condition for the fed state.

Chan et al. (31) observed that PDT with Chl*-a* metabolites completely inhibited the growth of hepatocellular carcinoma, while it did not affect normal hepatocytes. This observation is important because it agrees with the principle of PDT, according to which PSs selectively accumulate in the diseased tissue and neighboring vasculature, while their affinity for healthy cells should be low enough for them to be cleared from the healthy tissue (32).

It is believed that in healthy tissues the triplet state of the PS is eliminated by the defense mechanisms of the cell, which can, therefore, decrease the effects of the PDT (33,34).

In DM/PDT, the results obtained with the intact organ were similar to the acute results obtained in isolated cells, in which the Chl*-a* illumination reduced glucose release (*vs* controls) and glycogenolysis rate (*vs* DM) and stimulated glycolysis. The action exerted by Chl*-a* in both protocols with diabetic animals – reducing liver glucose metabolism – indicated a potential capacity of this PS in aiding the correction of glycemia. This, together with the high fluorescence observed inside those animals treated with the extract, indicated the possibility of increased permeation of the PS in the intestine and hepatic action similar to the direct action of the PS on the isolated hepatocytes. It is important to note that Chl*-a* PDT stimulated glycolysis in both experimental protocols with T1DM animals, a pathway known to be depressed in the diabetic condition in the absence of insulin (2). Koch and Deo (35) were the first to relate Chl as a nutritional supplement in the control of hyperglycemic conditions in T2DM. Their study, conducted *in vitro*, reported the inhibitory effect of Chl on the intestinal enzyme alpha-glucosidase, responsible for the digestion and absorption of dietary glucose. The authors suggested that inhibition of this enzyme could reduce the glucose content absorbed in such a way as to prevent postprandial peak glycemia and contribute to the maintenance of glucose homeostasis.

However, the results presented in Table 1 showed that treatment with CBE as a source of Chl*-a* in the absence of illumination did not affect postprandial glycemia nor the weight of the animals beyond the alteration caused by the diabetic condition itself. Therefore, the effects observed *in vitro* in T2DM (35) were not reproduced *in vivo* in experimental T1DM animals. In research conducted with *Caenorhabditis elegans* treated with Pheid, a metabolite of Chl*-a*, and light exposure, a reduction in fat mass was observed (36). However, this effect was only perceptible under illumination, which would explain the fact that in this study there was no change in body weight induced by treatment with CBE in the dark. Likewise, in the absence of photostimulation, the treatment did not cause changes in AST and ALT levels of the diabetic animals. These results imply the need of photoirradiation on PSs to stimulate their biological activity.

Important changes were also found in the evaluation of oxidative stress. The results showed that the treatment of diabetic animals with CBE alone was not enough to reduce oxidative stress in the liver. This was observed by the higher levels of carbonylated proteins and ROS, together with less activity of the antioxidant enzymes catalase and SOD in the homogenate of perfused livers in the absence of light stimulus. However, in livers perfused under photostimulation (DM/PDT), hepatic oxidative stress was reduced, as observed by lower levels of TBARS, carbonylated proteins, and ROS. Thus, PDT was beneficial in reducing the oxidative stress of the intact liver.

PDT states that, by photostimulation in the presence of oxygen, the PS can participate in the process of electron or energy transfer, forming free radicals or ROS (9). However, evaluation of ROS in this study revealed that light did not increase oxidative stress. In addition, levels of carbonylated proteins (which appear to be more adequate than TBARS to assess tissue oxidative damage, since they are more sensitive to both diabetes and the light stimulus) were reduced by 36% in DM/PDT compared to DM. This result corroborates those observed by Zhang et al. (36) in *Caenorhabditis elegans* treated with Pheid and exposed to illumination, in which an 80% reduction in carbonylated protein levels was detected.

Furthermore, the livers of DM animals presented higher levels of GSH than the livers of non-diabetic animals also treated with CBE and in the dark (CG), a result that apparently suggested that the treatment with the extract was only enough to raise GSH levels in diabetes. However, this increase was not sufficient to improve hepatic oxidative damage, which only occurred when the light stimulus was applied.

The results of this study are still insufficient to determine the mechanism by which liver photostimulation triggered an improvement in oxidative stress. However, it seems to be due to the stimulation of a part of the antioxidant system, that is, by the increase in catalase activity. Although there are reports that the direct antioxidant activity of Chl*-a* is low due to its high chemical instability (37), the results of this study indicated a promising antioxidant capacity of CBE stimulated by PDT.

The PDT applied to Chl*-a*, directly on hepatic cells and indirectly in the liver by oral administration of the Chl*-a*-rich extract, improved the general condition of the liver and of hepatocytes isolated from T1DM rats and, in this way, it is promising as an alternative and/or complementary treatment to T1DM. Unraveling the Chl*-a* mechanisms of action on the liver – possibly mediated by RXR agonism (13) – will improve the knowledge of these anti-diabetic effects of Chl*-a* (15).
